# 909. Small Feet, Hot Streets: Pediatric Pavement Burns in Desert Climates

**DOI:** 10.1093/jbcr/irag033.413

**Published:** 2026-04-06

**Authors:** Paul P Kim, Alexander Rowan, Vidhani Goel, Christopher Gonzalez, Brenna Chen, Kendra Coleman, Marianne Estrada, Kavita Batra, Rabia Nizamani, Joshua Khorsandi, Stephanie Martinez

**Affiliations:** Leon S. Peters Burn Center, Las Vegas, NV; Leon S. Peters Burn Center, Las Vegas, NV; Kirk Kerkorian School of Medicine at University of Nevada, Las Vegas, NV; Leon S. Peters Burn Center, Las Vegas, NV; Leon S. Peters Burn Center, Las Vegas, NV; Leon S. Peters Burn Center, Las Vegas, NV; Leon S. Peters Burn Center, Las Vegas, NV; Kirk Kerkorian School of Medicine at University of Nevada, Las Vegas, NV; UMC Lions Burn Care Center, Las Vegas, NV; Kirk Kerkorian School of Medicine at University of Nevada, Las Vegas, NV; University of Nevada, Las Vegas, NV

## Abstract

**Introduction:**

Pediatric contact burns pose unique challenges due to developmental vulnerabilities and risk for long-term sequelae. Among types of contact burns, pavement burns in desert climates, where ambient temperatures frequently exceed thresholds at which asphalt can rapidly cause contact burns, are a rare but morbid injury in children. Our study characterizes patterns, hospital course and outcomes of pediatric contact burns, with emphasis on pavement-related injuries.

**Methods:**

A retrospective review was conducted of patients <18 years admitted to a regional burn center from 2014–2023 with documented contact burns. Demographic, injury, and outcome variables were compared between pavement and non-pavement burns using t-tests and chi-square tests, with significance set at *p*<.05.

**Results:**

Ninety-eight pediatric contact burn cases were identified, including 20 (20.4%) pavement burns. Patients with pavement burns were older on average (5.6 ± 6.0 vs. 4.0 ± 4.9 years, *p*=.315) but not statistically different. Pavement burns were highly seasonal, with 90% occurring in summer compared to 36% of other contact burns (*p*<.001). Pavement burns were associated with larger TBSA (4.2 ± 3.7% vs. 2.2 ± 2.4%, *p*=.041), higher mortality (5.0% vs. 0%, *p*=.047), more frequent ICU admission (37.5% vs. 6.9%, *p*=.025), and increased ventilator use (10.0% vs. 0%, *p*=.005). Mean hospital length of stay did not differ significantly (4.1 ± 5.1 vs. 4.7 ± 6.1 days, *p*=.644).

**Conclusions:**

Pavement burns, though less common than other contact burns in children, are associated with significantly greater injury severity, ICU needs, and mortality. These findings highlight the need for targeted prevention in high-temperature environments, particularly in summer months, and early recognition of pavement burns as high-risk injuries in children.

**Applicability of Research to Practice:**

Findings support the development of community-level interventions in high-temperature regions to design awareness campaigns and safety interventions to serve as effective, low-cost strategies to reduce pediatric pavement and other contact burn incidence.

**Funding for the study:**

N/A.

